# Challenges and Opportunities towards the Development of Risk Assessment at the Consumer Phase in Developing Countries—The Case of *Campylobacter* Cross-Contamination during Handling of Raw Chicken in Two Middle Eastern Countries

**DOI:** 10.3390/pathogens9010062

**Published:** 2020-01-16

**Authors:** Ihab Habib, Ali Harb, Ingrid Hansson, Ivar Vågsholm, Walaa Osama, Salma Adnan, Mohamed Anwar, Neveen Agamy, Sofia Boqvist

**Affiliations:** 1 Veterinary Medicine Department, College of Food and Agriculture, United Arab Emirates University (UAEU), P.O. Box 15551, Al Ain, UAE; 2 High Institute of Public Health, Alexandria University, Alexandria 21568, Egypt; w.osama2008@yahoo.com (W.O.); dr.salmaegypt@yahoo.com (S.A.); drmohamedanwar1@gmail.com (M.A.); 3 School of Veterinary Medicine, Murdoch University, Perth 6150, Australia; Ali.Harb@murdoch.edu.au; 4 Thi-Qar Public Health Division, Ministry of Health, Thi-Qar, Iraq; 5 Department of Biomedical Science and Veterinary Public Health, Swedish University of Agriculture Science, SE 750 07 Uppsala, Sweden; ivar.vagsholm@slu.se

**Keywords:** *Campylobacter*, Middle East, risk assessment, Egypt, Iraq

## Abstract

In many low- and middle-income countries, data limitations are a major challenge facing the development of food safety risk assessment. In the present study, a questionnaire data collection tool was designed with an emphasis on gathering specific data points required by a risk modeller for simulating a scenario of *Campylobacter* cross-contamination during handling of raw chicken meat at the consumer phase. The tool was tested in practice to support its value and applicability in settings where data limitations are a challenge. The study subjects were 450 consumers in two Middle Eastern settings: Alexandria in Egypt (*n* = 200) and Thi-Qar in Iraq (*n* = 250). The majority (78.5%) of respondents in Egypt opted for wet markets/live bird shops as their preferred source of chicken meat. In contrast, 59.6% of Iraqi respondents preferred to buy chicken meat from supermarkets. Added to that, 73.0% of consumers in Egypt and 56.8% of consumers in Iraq viewed the quality of frozen chicken as “inferior” to that of chicken from wet markets. Almost all respondents in both Egypt and Iraq shared the practice of washing chicken in water before cooking. The percentage of consumers who ‘very frequently’ or ‘frequently’ prepare chicken prior to making the salad was 32.5% and 55.2% in Egypt and Iraq, respectively. A sizeable proportion of respondents in Iraq (40.8%) reported that they did not consider washing their hands with soapy water after touching raw chicken and preparing a salad in their home kitchen. Finally, 28.8% and 6.5% of respondents in Iraq and Egypt, respectively, indicated that they would not consider using a separate cutting board to avoid cross-contamination between raw chicken and salad. The data collection tool used in this study was designed in the first instance to match a conceptualised risk assessment framework, and that enabled the simultaneous collection of data points on consumption frequency, serving sizes, purchasing patterns, retail chain diversity and food handling practices. Results from such study design could be used for future development of a quantitative risk assessment model and to support food safety promotion efforts for domestic consumers in two of the most populated Middle Eastern countries.

## 1. Introduction

Poor food handling and hygiene practices in home kitchens are thought to be a significant cause of foodborne illness. Through poor handling practices, domestic food handlers can negate many of the benefits of food processing in producing safe food [1]. Improper handling of raw poultry meat in the home kitchen plays a significant role in human exposure to foodborne pathogens; notably *Salmonella* and *Campylobacter* [2]. Quantitative risk assessment studies, some of which have focused primarily on *Campylobacter* transfer at the consumer phase, have been carried out in a number of countries to develop strategies to reduce the risk of infection [3]. Assessment of exposure at the consumer phase is of interest because it is less controlled compared with other stages in the food chain. In addition, it is crucial to understand the frequency of consumption and the degree to which foods are becoming contaminated at the consumer phase because these directly influence the risk characterisation estimate [2,3]. Since food handling is an essential for the preparation of safe food, increasing knowledge about the likelihood of cross-contamination at the consumer level is required for the development of exposure assessments models [4].

Modelling can facilitate the quantification of food safety risk. Once a consumer phase model has been developed, the effects of various control strategies and trends may be simulated [2]. However, quantitative risk assessment modelling can be challenging because of its strong reliance on data. Published risk assessments on *Campylobacter*, including those developed by international organisations, are based mostly on data from high-income countries [3]. Few attempts have been made to build consumer phase models for *Campylobacter* in chicken meat in low- and middle-income countries [2,5]. The data gap in the prevalence and level of *Campylobacter* contamination of chicken for assessing risk is well known in these settings. Additionally, the lack of data on consumption and purchase patterns, preparation practices and cross-contamination likelihood has hindered the development of quantitative risk assessment models in low- and middle-income countries [6].

The World Health Organization (WHO) report on the global burden of foodborne diseases concluded that infection with *Campylobacter* is among the leading bacterial causes of enteric illnesses in the Middle East, comparable to reported *Campylobacter* infection in sub-Saharan Africa and south-east Asia regions [7]. Egypt and Iraq are among the most populated countries in the Middle East, and their food cultures are representative of those of many Arabic and Middle Eastern countries. In addition, Egypt and Iraq face the sociopolitical challenges of many developing nations, including a fragile political environment, an influx of refugees and poor sanitation, food safety, and security [8]. Public health systems in Egypt and Iraq struggle to respond to the evolving burden of enteric illnesses because they lack surveillance of human exposure to important enteric pathogens such as *Campylobacter* in their respective food systems. There is a limited number of baseline studies investigating *Campylobacter* epidemiology at the human-chicken meat interface in Egypt and Iraq. In 2016, a study in Alexandria (Egypt) concluded that 61.9% of the tested chilled chicken meat were positive for *Campylobacter jejuni*, and the pathogen was also detected in 66.7% of the surveyed chicken handlers and symptomatic consumers [9]. In Thi-Qar (Iraq), a recent study pointed that *Campylobacter* spp. (17/155 (10.9%)) were the third most common pathogen to be isolated from diarrhoeal children aged less than 5 years old [10]. Hence, with the objective of developing a quantitative consumer phase risk assessment model for *Campylobacter*, this study aims to fill the gap in the knowledge regarding the variability in consumer purchase preferences, frequency of consumption of raw chicken meat and cross-contamination practices when handling raw chicken in two settings, Alexandria (Egypt) and Thi-Qar (Iraq). This work is part of a larger research project and was done in parallel with ongoing baseline microbiological surveys of retail chicken meat in both countries.

## 2. Results

### 2.1. Profile of Respondents

**Figure 1:**
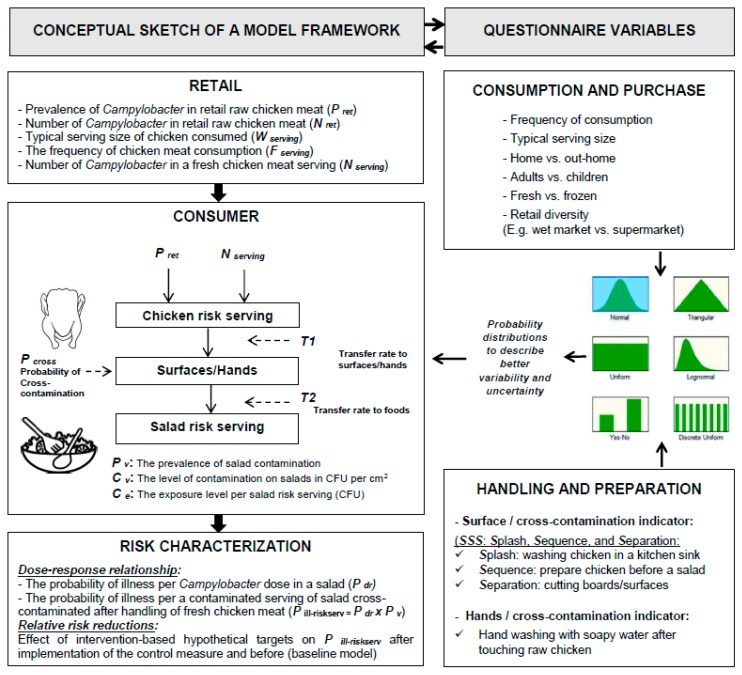
Conceptual framework for a model of consumer phase risk assessment (to the left). The questionnaire data collection tool (to the right) was tailored to characterize the key input variables required for building up the model framework.

A questionnaire data collection tool was designed to generate the parameters needed to inform the development of a *Campylobacter* quantitative risk assessment. The data collection instrumentation was based on a “conceptual framework” (Figure 1) of a hypothetical consumer phase risk assessment model; for such hypothetical model a questionnaire was tailored to inform the key variables needed by a risk assessor. A total of 450 questionnaires were obtained from the two study settings in Egypt (*n* = 200) and Iraq (*n* = 250) as shown in Table 1. Most respondents in both settings were female, reflecting the ratio of people responsible for preparing food in Egyptian and Iraqi households. The younger (<35 years) and ever married groups were well represented in both settings (Table 1). Education levels varied, but the frequency of respondents who had attained tertiary qualifications was significantly higher (*p* < 0.05) in respondents from Alexandria (Egypt) compared with those from Thi-Qar (Iraq).

### 2.2. Chicken Meat Consumption and Purchasing Patterns

Respondents were asked about their frequency of chicken consumption per week. Of the respondents from Egypt, 45.5% indicated that chicken was consumed twice weekly at home, while 31.5% ate chicken outside the home once a week (Table 2). In contrast, a higher proportion of Iraqi respondents ate chicken outside of the home (48.0% once weekly and 36.0% twice weekly) (Table 2). In an attempt to characterise a typical serving size, respondents were asked to describe the most likely amount of chicken meat consumed in one sitting by adults and children in the household. For adults, a quarter of a chicken was the most likely serving size in both Egypt (84.5%) and Iraq (58.0%) (Table 2), although 26.0% of Iraqi respondents said that adults were most likely to consume less than a quarter of a chicken. As expected, the most likely serving size of chicken for children was less than a quarter of a chicken in both settings (Table 2).

Generating data on consumer preferences for buying chicken from formal vs. informal markets and the proportion of fresh vs. frozen chicken purchased was essential for the development of the quantitative risk assessment model is the study settings. Hence, respondents were asked about their preferred sources and presentation of purchased chicken meat (Table 2). The majority (78.5%) of respondents in Egypt opted for wet markets/live bird shops as their preferred source of chicken meat. In contrast, 59.6% of Iraqi respondents preferred to buy chicken meat from supermarkets. This variation was further elaborated when respondents were asked to nominate their preferred presentation of purchased chicken meat. Most Iraqi consumers (58.8%) preferred to buy frozen chicken meat, which is typically found in supermarkets only. A whole chicken carcass was the most popular form purchased by consumers in both settings (Table 2). Respondents were asked about their perceptions of frozen chicken meat. Interestingly, 73.0% of consumers in Egypt and 56.8% of consumers in Iraq viewed the quality of frozen chicken as “inferior” to that of chicken from wet markets. Among the Iraqi respondents, 27.6% perceived frozen chicken to be of better quality than chilled chicken (Table 2).

### 2.3. Chicken Meat Handling and Preparation Practices

Questions about practices (Table 3) of food handling and preparation were asked to generate responses that could be converted to probability distributions to describe key cross-contamination events in home kitchens in each of the study settings. The data shown in Table 3 reveal that almost all respondents in both Egypt and Iraq shared the practice of washing chicken in water before cooking, with 40% of respondents in Iraq tending to wash chicken meat directly in the kitchen sink under cold running tap water and a similar 39.2% of Egyptian respondents tending to wash chicken under cold running tap water in a cooking pot rather than directly in the kitchen sink. Most (95.6%) Iraqi consumers indicated they used water only, while 56.0% of Egyptian respondents used additional ingredients, either alone or in combination, for washing chicken meat, including salt, flour, lemon and soap (Table 3).

To better understand the cross-contamination pathways in home kitchens, consumers were asked to respond to a scenario involving the preparation of a meal consisting of chicken and a salad. The percentage of consumers who ‘very frequently’ or ‘frequently’ prepare chicken prior to making the salad was 32.5% and 55.2% in Egypt and Iraq, respectively (Table 3). There was a highly significant variation (*p* < 0.0001) between the two settings in reported answers on handwashing after touching raw chicken—while 90.4% of respondents in Egypt indicated they washed their hands with soapy water, only 27.2% of respondents in Iraq indicated doing so. A significant (*p* < 0.05) proportion of respondents in Iraq (40.8%) reported that they did not consider washing their hands with soapy water after touching raw chicken and preparing a salad in their home kitchen. Finally, 28.8% and 6.5% of respondents in Iraq and Egypt, respectively, indicated that they would not consider using a separate cutting board to avoid cross-contamination between raw chicken and salad.

## 3. Discussion

The primary purpose of this research was to provide data that could be used for future development of a quantitative risk assessment model and to support food safety promotion efforts for domestic consumers in two of the most populated Middle Eastern countries. More detailed information was gathered on chicken consumption and purchasing patterns than on chicken handling and preparation practices because of the well-accepted fact that self-reported surveys tend to overestimate standard kitchen hygiene and do not always reflect actual consumer practices [11,12]. Consequently, data on handling and preparation practices were reported in general terms only, with an emphasis on specific data points that might be needed by a risk modeller for simulating *Campylobacter* cross-contamination during handling of raw chicken meat.

For low- and middle-income countries to establish their own quantitative risk assessments, the general frameworks of risk assessment applied to high-income countries may be useful, but data extrapolation may not be always valid because of the vast differences in consumption, purchasing and consumer practices, which are crucial for precise risk estimates [2]. In many low- and middle-income countries, data limitations, including lack of availability or data being unfit for purpose, has made the development of risk assessment models challenging [6,13]. The present study generated valuable data from Egyptian and Iraqi contexts that may be applied to other settings in the Middle East given the similarities in their poultry retail chains. The data collection approach adopted in this study was concise and inexpensive and may be easily applied in other countries. The present study gathered information regarding frequency of chicken meat consumption and noted some variation between the two study settings (Table 2). Data on consumption patterns (i.e., serving size and frequency of consumption) among populations is crucial for assessing public health risks associated with the prevalence and concentration of foodborne pathogens (e.g., *Campylobacter*) in retail chicken meat [14,15]. The questionnaire instrument used in the present study was designed so that responses could be easily translated into probability distributions describing variability in consumption frequency. For instance, data can be described using a discrete distribution, which is typically used to define variables that can take one of several explicit discrete values (*x*) and for which probability weights (*p*) are assigned to each value [16]. Probability distribution parameters for serving sizes were intended to determine the pathogen dose ingested per meal, i.e., the number of colony forming units (CFU/g) x serving size (g). As expected, our results revealed considerable variations in serving size between subpopulations (i.e., adult vs. children); thus, serving sizes generated in the present study could be pooled or separated using the fitted probability distribution for each subpopulation, depending on the scope of the risk assessment [17].

In contrast to high-income countries, wet market/live bird shops play an important role in supplying chicken meat to consumers in many low- and middle-income countries [18]. Hence, it was important to assess the ratio of wet market to supermarket chicken in both settings. This was also crucial to inform the development of risk assessment by weighting such ratios against the results from microbiological surveys of chicken sampled from each retail chain. Interestingly, consumers in Egypt opted to purchase much of their chicken meat from the wet market. Conventional wet markets are popular as they provide live chickens slaughtered onsite but have short operating hours (~6 h/day) in the morning. This ensures that the chicken sold each day is fresh. Some studies have indicated that *Campylobacter* contamination of chicken from wet markets is comparatively higher than that of chicken from supermarkets, while others have concluded the opposite [19,20].

In Iraq, a significant proportion of respondents favoured frozen chicken purchased from supermarkets over fresh chicken purchased from wet markets. The importation of frozen chicken meat has steadily increased in Iraq over recent years because the considerable increase in domestic production has failed to keep pace with growing demand (United States Agency for International Development (USAID), 2006) [21]. Maintaining chilled food products in many Iraqi retail stores can be challenging, especially during the long summer months, because of frequent electricity outages arising from decaying infrastructure and prolonged heat waves. Hence, the growth in consumer demand for frozen chicken in Iraq could be attributable to it being less susceptible to spoilage compared with chilled products [22]. Although there is no growth of *Campylobacter* during refrigeration or freezing of chicken meat, freezing has been shown to reduce the number of *C. jejuni* up to 2.9 log, while refrigeration has a negligible effect [23]. Conditions leading to a decrease in *Campylobacter* counts could lower the risk to consumers—a Danish risk assessment estimated that a decrease of 2 log_10_ CFU/g could lead to a 30-fold reduction in the human incidence of *Campylobacter* infection [24]. In low- and middle-income countries, data on the diversity and complexity of retail supply chains together with a reasonable analysis of consumer purchase preferences are essential for developing reliable food safety risk assessment.

With the aim of developing a consumer phase risk assessment for *Campylobacter*, the present study focused on selected cross-contamination indicators during chicken meat handling and preparation in the home kitchen. *Campylobacter* species are heat sensitive and cannot multiply or grow below 30 °C. However, multiplication in the food is not necessary as the infectious dose for campylobacteriosis is low (~500 CFU) [25]. Therefore, cross-contamination during food preparation is the most critical food handling risk factor for promoting the development of human infection [4,15]. Our results revealed some concerning consumer practices that could promote cross-contamination during handling and preparation of raw chicken meat. A total of 99.5% and 97.2% of respondents from Egypt and Iraq, respectively, indicated that they washed raw chicken before cooking. In both settings, almost half the respondents reported they typically washed raw chicken with water in the kitchen sink. In contrast, a study across different Asian countries showed that only 75% of surveyed consumers in India, 46% in Korea and 48% in Thailand reported washing raw poultry before cooking [26]. Food safety experts recommend that consumers should not wash or rinse raw poultry because splashing of contaminated water may transfer pathogens to other foods and kitchen surfaces. One study found that contaminated water from washing raw poultry can travel up to 28 inches (71 cm) on either side of the sink and 20 inches (51 cm) in front of the sink [27]. Several studies have confirmed the capability of *Campylobacter*, more than any other pathogen, to spread quickly and easily on surfaces and to cause contamination and consequent infection if ingested by consumers [28,29].

Our results showed that 44% of respondents in Egypt used other ingredients in the washing of chicken, including vinegar, salt, flour, lemon and soap. The effect of the addition of these ingredients on bacterial pathogens harbouring the surface of chicken meat is difficult to evaluate given the variations in contact times and amounts used by different consumers [30].

Commonly quoted qualitative responses (open-ended questions) from the Egyptian survey regarding the washing of chicken before cooking included ‘it is recommended by cookbooks or cooking shows’, ‘a practice taught from mother to daughter’, ‘to mask unwanted smell or sliminess from the source’ or ‘I have my routine’. The more often tasks such as washing chicken are repeated, the more automatic they become and the less cognitive effort is needed; therefore, it may be challenging to find interventions to break the habit [31]. Additionally, some household food preparers felt that the introduction of new behaviours such as not washing chicken before cooking may diminish the opinions of others about their cooking skills/abilities.

In some published studies on quantitative risk assessment of human campylobacteriosis, salad was chosen as a food at risk of cross-contamination during handling of raw chicken meat in home kitchens [32,33]. Hence, in the current study, we focused on obtaining responses that could inform the probability of cross-contamination between raw chicken meat and salad in poor practice scenarios. Our results show that almost half the respondents in Iraq and a third of respondents in Egypt indicated that they typically prepared chicken before preparing salad in their home kitchens (Table 3). Additionally, 40.4% and 63.5% of respondents in Iraq and Egypt, respectively, indicated that they would consider using separate cutting boards for raw chicken and salad (Table 3). Our results on consumer habits showed that many consumers in the two study settings prepared their food in such a way that could supports the transfer of microorganisms from raw chickens to ready-to-eat foods. Mylius et al. [34] identified that the risk of acquiring *Campylobacter* infection is positively related to the preparation of raw chicken prior to preparation of salad, and negatively related to handwashing and washing of cutting boards following preparation of raw chicken. This information should be considered in the design of public campaigns to improve food hygiene practices, which should emphasise the use of separate cutting boards or the washing of cutting boards and the importance of not preparing raw meat prior to the preparation of ready-to-eat foods.

The present study noted that handwashing with soap and water after touching raw chicken was the favoured approach for 90.4% of respondents in Egypt, while only 27.2% of Iraqi respondents indicated that they did so. In comparison, Koppel and colleagues reported that handwashing with soap and water after touching raw chicken was a reported favoured practice for more than half of surveyed consumers in India and Thailand and a third of consumers in Korea [26]. It is important to note that results from self-reported consumer practice surveys should be treated with caution because respondents frequently report what is perceived to be right rather than what they actually do [11]. To overcome such limitation, future studies should be undertaken to conduct observational studies at the consumer level in order to assess more accurately the prevalence of unsafe handling practices.

## 4. Material and Methods

### 4.1. Study Design and Settings

A cross-sectional study of the frequency of purchasing, consumption and handling of raw chicken was conducted from June to December 2017 in two Middle Eastern cities—Alexandria, the second largest city in Egypt after Cairo, and Thi-Qar, considered the least developed governorate in Iraq [35,36]. Both settings are densely populated and hence considered suitable for providing insight about complex food systems utilised by a diversity of consumers. These cities represent typical Middle Eastern settings in the variability of exposure routes for human *Campylobacter* infection such as the purchase of local vs. imported or fresh vs. frozen chicken from informal vs. formal markets. This investigation comprises part of a study aimed at developing a quantitative risk assessment for foodborne pathogens commonly associated with chicken meat in Middle Eastern and African settings.

### 4.2. Instrumentation

A questionnaire data collection tool was designed to generate the parameters required to inform the development of a *Campylobacter* quantitative risk assessment. The data collection instrumentation was designed as follows: First, we constructed a “conceptual framework” (Figure 1) for a hypothetical consumer phase risk assessment applicable to the study settings. Second, we tailored the questionnaire to inform the key variables needed by a risk assessor. Thus, we deviated from the traditional format for collecting data on food safety knowledge, attitudes, and practices to a format driven by the risk assessment framework. A 20-item written questionnaire was designed and pilot tested by ten participants (in each setting) prior to the survey to confirm question clarity and to identify participants’ opinions and time requirements. The pilot trial resulted in minor modifications to the wording of questions. The questionnaire was written in English and translated to Arabic by a native researcher from each country, before being translated back to English to verify the clarity of translation. The final revised questionnaire was divided into four sections: (1) demographic characteristics, (2) chicken meat consumption, (3) chicken meat purchase, and (4) chicken meat handling and preparation practices (the questions are presented through Table 2 and Table 3). Each questionnaire took ~25 min to administer. Data were collected on weekday mornings and afternoons when a member of the target group would most likely be at home.

### 4.3. Data Collection and Analysis

The study protocols were reviewed and approved by the Murdoch University Human Research Ethics Committee (Permit No. 2015/224). The inclusion criteria for the target group were that interviewees be at least 18 years of age and be primarily responsible for the household food preparation. Voluntarily permission to participate was sought. The aims and objectives of the study were explained to each interviewee and confidentiality of their information was confirmed verbally.

Respondents were randomly selected from households in Alexandria (Egypt) and Thi-Qar (Iraq). In Alexandria, the investigators and five trained postgraduate university students (in the field of Nutrition and Public Health sciences) visited each household and explained the purpose and nature of the study to interviewees. The household survey was a component of Alexandria University’s High Institute of Public Health postgraduate capstone unit (Public Health Surveillance) for which students were required to conduct an annual survey on public and environmental health in a selected suburb (representing east, west, and centre of the city).

In Thi-Qar, the investigators and a team of 12 trained public health nurses (from Thi-Qar Public Health Division) executed the household survey using the same questionnaire and in a similar fashion to that in Alexandria. The survey took place in conjunction with a door-to-door household polio vaccination campaign by the Iraqi Ministry of Health.

Information collected from completed questionnaires was entered into Microsoft Excel 2010, and Stata 11.0 software was used for descriptive data analysis [37]. Frequencies and percentages of responses in each category were calculated and presented in tabular form. Chi-square tests were performed to test the relationship between variables of interest.

## 5. Conclusions

A questionnaire data collection tool was designed and tested in practice to evidence its value in generating some of the key specific parameters required to inform the development of a *Campylobacter* consumer phase quantitative risk assessment model. This tool was simple, inexpensive and easily applied in low- and middle-income countries where data limitations have hindered the application of risk assessment to tackle local food safety challenges. The instrument used in this study allowed the simultaneous collection of data on consumption frequency, serving sizes, purchasing patterns, retail chain diversity, and food handling practices. It should be noted that risk assessment as an approach requires a simplified representation, based on assumptions and hypotheses, of a complex reality. Thus, the conceptualised framework that guided the development of the data collection instrument used in this study should not be viewed as a static framework but as an interactive tool that can be better refined with more questions and details relevant to settings wherever it will be used. Laws and regulations cannot enforce controls and measures to prevent unsafe practices at home, as is the case for commercial food processors and retailers. The only possibility for changing risky practices in private home kitchens is consumer education.

## Figures and Tables

**Table 1:** Descriptive characteristics of the consumers.

Demographic Characteristics	Egypt (*n* = 200) Frequency (%)	Iraq (*n* = 250) Frequency (%)
**Gender**		
Male	40 (20.0)	19 (7.6)
Female	160 (80.0)	231 (92.4)
**Age group**		
<35	107 (53.5)	150 (60.0)
35–50	40 (20.0)	85 (34.0)
>50	53 (26.5)	15 (6.0)
**Marital status**		
Single	24 (12.0)	18 (7.2)
“Ever-married” (married, divorced, widowed)	196 (88.0)	232 (92.8)
**Highest educational level**		
Illiterate	4 (2.0)	18 (7.2)
Primary level	40 (20.0)	40 (16.0)
Secondary level	96 (48.0)	160 (64.0)
Tertiary level *	60 (30.0)	32 (12.8)

* Tertiary education refers to any type of education pursued beyond the secondary (high school) level.

**Table 2:** Chicken meat consumption and purchasing patterns among respondents.

Questions	Egypt (*n* = 200) Frequency (%)	Iraq (*n* = 250) Frequency (%)
Over the last week, how many times did you eat chicken meat at your home?		
0	9 (4.5)	3 (1.2)
1	54 (27.0)	46 (18.4)
2	91 (45.5)	93 (37.2)
3	31 (15.5)	78 (31.2)
4	14 (7.0)	27 (10.8)
>4	1 (0.5)	3 (1.2)
Over the last week, how many times did you eat chicken meat outside the home (e.g., in a restaurant or delivery)?		
0	123 (61.5)	1 (0.4)
1	63 (31.5)	120 (48.0)
2	11 (5.5)	90 (36.0)
3	2 (1.0)	38 (15.2)
4	1 (0.5)	1 (0.4)
>4	-	-
For an adult member (≥18 years) in your family, which of the following describes better the most likely amount of chicken meat consumed per one eating occasion?		
Less than ¼ of a chicken	15 (7.5)	65 (26.0)
¼ of a chicken	169 (84.5)	145 (58.0)
½ of a chicken	13 (6.5)	38 (15.2)
Whole chicken	-	2 (0.8)
For a child (<18 years) member in your family, which of the following describes better the most likely amount of chicken meat consumed per eating occasion? *		
Less than ¼ of a chicken	61 (54.0)	216 (86.4)
¼ of a chicken	48 (42.5)	34 (13.6)
½ of a chicken	2 (1.7)	-
Whole chicken	1 (0.8)	-
Where do you purchase chicken meat?		
Wet markets/live bird shops	157 (78.5)	101 (40.4)
Supermarkets	39 (19.5)	149 (59.6)
Others	Home-raised: 2 (1.0) Direct from farm: 2 (1.0)	-
What is the preferred display presentation of your purchased chicken meat?		
Freshly slaughtered on site, from wet markets **/live bird shops	163 (81.5)	103 (41.2)
Chilled, from cold supermarket store	25 (12.5)	-
Frozen, from a supermarket freezer	12 (6.0)	147 (58.8)
What is the preferred form of your purchased chicken meat?		
Whole carcass	141 (70.5)	179 (71.6)
Portions with skin (e.g., drumstick)	19 (9.5)	34 (13.6)
Portions without skin (e.g., fillet)	40 (20.0)	47 (14.8)
What is your perception of frozen chicken meat?		
Better in quality than chilled ones	13 (6.5)	69 (27.6)
Inferior in quality than chilled ones	21 (10.5)	29 (11.6)
Better in quality than those from the wet market	11 (5.5)	10 (4.0)
Inferior in quality than those from the wet market	146 (73.0)	142 (56.8)
Inferior in quality than both of chilled and wet market	9 (4.5)	-

* Data from Egypt: The descriptive result was provided for 113 respondents only, as 87 indicated having no children; ** Wet market is defined as a traditionally places that sold on-site slaughtered and live food animals out in the open, among many other commodities.

**Table 3:** Chicken meat handling and preparation practices among respondents.

Questions	Egypt (*n* = 200) Frequency (%)	Iraq (*n* = 250) Frequency (%)
In your home kitchen, do you wash chicken with water before cooking?		
Yes	199 (99.5)	243 (97.2)
No	1 (0.5)	7 (2.8)
If yes in the previous question, how do you wash chicken with water before cooking?		
Running cold tap water in a kitchen sink	65 (32.6)	97 (40.0)
Running hot tap water in a kitchen sink	18 (9.4)	26 (10.7)
Running cold tap water in a cooking pot	78 (39.2)	48 (19.7)
Running hot tap water in a cooking pot	38 (19.1)	72 (29.6)
Do you use anything other than water to wash chicken before cooking?		
Yes	88 (44.0)	239 (95.6)
No	112 (56.0)	11 (4.4)
If yes in the previous question, please mention the thing(s) other than water that you use to wash chicken before cooking?		
	Vinegar: 16 (18.2)	Soap: 8 (72.7)
	Vinegar + salt + flour: 16 (18.2)	Vinegar: 3 (27.3)
	Salt: 8 (9.1)	
	Salt + vinegar: 8 (9.1)	
	Salt + lemon + flour: 8 (9.1)	
	Soap: 4 (4.5)	
	Other combinations: 28 (31.8)	
How frequently do you prepare chicken before preparing a salad meal?		
Very frequently	40 (20.0)	75 (30.0)
Frequently	25 (12.5)	63 (25.2)
Occasionally	60 (30.0)	45 (18.0)
Rarely	33 (16.5)	25 (10.0)
Never	42 (21.0)	42 (16.8)
While preparing a salad meal along with chicken, do you consider washing your hands with soapy water after handling raw chicken? *		
Yes, I take that into consideration	179 (90.4)	68 (27.2)
Sometimes yes and sometimes no	16 (8.1)	80 (32.0)
No, I do not take that into consideration	3 (1.5)	102 (40.8)
While preparing a salad meal along with chicken, do you consider using separate cutting boards for preparation and cutting each of them?		
Would not consider	13 (6.5)	27 (28.8)
Might or might not consider	60 (30.0)	77 (30.8)
Definitely consider	127 (63.5)	101 (40.4)

* Data from Egypt: Descriptive results are provided for 198 respondents only, with two missing answers.
